# Water-dispersable photoreactors based on core–shell mesoporous silica particles

**DOI:** 10.1038/s41598-024-61750-8

**Published:** 2024-05-17

**Authors:** Andrzej Baliś, Dominika Lorens, Arkadiusz Gut, Szczepan Zapotoczny

**Affiliations:** 1 Faculty of Chemistry, Jagiellonian University, Gronostajowa 2, 30-387 Krakow, Poland; 2 Jerzy Haber Institute of Catalysis and Surface Chemistry, Polish Academy of Sciences, Niezapominajek 8, 30-239 Krakow, Poland

**Keywords:** Mesoporous silica nanoparticles, Fluorescence resonance energy transfer, Photoreactors, Co-condensation, Photooxidation, Nanoreactors, Nanoparticle synthesis, Photocatalysis, Energy transfer

## Abstract

Robust solid-core silica particles with submicrometer size and anthracene-containing mesoporous shell were obtained and studied as model water-dispersable photoreactors. An anthracene derivative containing a triethoxysilyl group was synthesized and co-condensed with tetraethoxysilane in various ratios to form a photoactive mesoporous shell with a thickness up to approximately 80 nm on previously prepared solid silica particles. Mesopores of as-synthesized particles, without a commonly applied removal of the micellar templates, offered a confined space for solubilization of hydrophobic molecules. Efficient excitation energy transfer from anthracene chromophores to both hydrophobic (perylene) and hydrophilic (fluoresceine) encapsulated acceptors was observed in an aqueous dispersion of the particles. Photosensitized oxidation of encapsulated perylene was shown to proceed efficiently in such systems serving as water-dispersable photoreactors. Importantly, the designed core–shell systems were found to be stable for a long time (at least 24 months) and robust enough, thanks to the presence of solid cores, to be handled by centrifugation in aqueous dispersions. All these features make them promising candidates for reusable systems for the photosensitized degradation of water pollutants, especially hydrophobic pollutants.

## Introduction

Mesoporous silica nanoparticles (MSNs), since their invention^1,2^, have continued to be widely studied and applied due to their useful properties, such as adjustable pore size^3^, diverse morphology^4^ large surface area and ease of modification during^5^ and after^6^ their synthesis. Soft-templating synthesis of MSNs is a facile and effective fabrication method, leading to a well-defined pore structure and particle size control^7^ Such porous structure, which is obtained by growing a silica network on micellar rods of surfactants, makes this kind of material attractive for applications in selective adsorption^8,9^ catalysis^10,11^ sensing^12^ drug delivery systems^13–15^, etc.

The development of a high specific surface area that gives MSNs its desired sorption properties is achieved by calcination or extraction of templates from the silica network. Without the removal of template agents, such raw, as-synthesized MSNs seem to be less applicable due to their low surface area. However, the micellar confined phases in the mesopores may also be utilized, e.g., controlled release of active substances^16^, capturing CO_2_^17^ or pollutants such as benzene and phenol^18^, opening new application fields for the as-prepared particles. Various systems offering a confined space may increase the yield of reactions proceeding therein, compared to bulk systems^19^, as has been shown, e.g., photodimerization of acenaphthylenes^20^ enzymatic reactions^21^ and hydrogen production^22^.

The co-condensation in the case of porous silicas is a process in which organically modified silica phases are obtained during one-pot synthesis. Synthesis of mesoporous silica modified with various organic groups have been reported and applied^23,24^. It is worth noting that the organic groups covalently incorporated into silica network typically remain intact after the removal of the surfactant via extraction. Thanks to unique mesoporous structure, tunable framework composition and low toxicity, mesoporous organosilica nanoparticles can be applied as e.g., sensors for diverse therapeutic or diagnostic applications as well as systems for efficient removal of nitrites or Pb^2+^ from water^25,26^. However, to the best of our knowledge, there are no reports on the application of raw MSNs as water-dispersable reactors, especially photoreactors, hosting hydrophobic reactants.

The micellar confined spaces in mesopores of raw silica particles dispersed in water are suitable for solubilization of hydrophobic compounds^27^, similar to microheterogeneous systems serving as nanocarriers/nanoreactors formed by e.g., amphiphilic polyelectrolytes in water^28,29^ or deposited on surfaces^30^. In such systems, once they are equipped with photoactive groups, efficient fluorescence resonance energy transfer (FRET) may occur, which is important for imaging^31^ and sensing applications^32,33^, photodynamic therapy^34^ and fabrication of water-dispersable photoreactors offering spatial confinement. MSNs are transparent in a broad UV‒Vis range so they found applications as luminescent biomarkers^35^ but they offer also a relatively large loading capacity and both those features very attractive for photoreactors applications. While all-mesoporous particles may exhibit limited mechanical stability^36^, growing a mesoporous shell on a solid support should bring mechanical robustness high enough to enable their separation from the reaction mixture using simple centrifugation.

Herein, we developed solid core mesoporous shell (SCMS) silica particles with anthracene (An) chromophores immobilized in the mesoporous shell. These materials may serve as water-dispersable microphotoreactors suitable for the realization of photosensitized reactions within the confined mesoporous environment. Such new systems were shown to host both hydrophilic and hydrophobic molecules in mesopores filled with cylindrical micelles. In these materials, efficient FRET occurring from An donors to perylene (Pe) or fluorescein (Fl) acceptors was demonstrated, and photosensitized oxidation of Pe served as a model photoreaction in such a confined environment.

## Experimental section

### Materials

Tetraethoxysilane (98%) (GC) (TEOS), hexadecyltrimethylammonium bromide (98%) (CTAB), dibutyltin dilaurate (95%), 3-(triethoxysilyl)propyl isocyanate (95%), fluorescein sodium salt (Fl, > 97,5%), and hydrochloric acid ACS reagent (37%) were purchased from Sigma Aldrich (USA). 9-(Hydroxymethyl)anthracene (> 98%) was purchased from TCI (Belgium). Perylene (Pe, 96%) was purchased from Koch-Light Laboratories (United Kingdom). Phosphate buffer saline (PBS) buffer solution was prepared from a tablet purchased from Sigma Aldrich. One tablet dissolved in 200 mL of deionized water yields 0.01 M phosphate buffer, 0.0027 M potassium chloride and 0.137 M sodium chloride (pH = 7.4 at 25 °C). Sodium carbonate was purchased from Avantor Performance Materials S.A. (Poland). Ammonia solution (30%, p.a.), ethanol (96%, p.a.), tetrahydrofuran (p.a.), dichloromethane (p.a.) were purchased from Chempur (Poland). All reagents were used as received without purification. Deionized water was used in all procedures.

### Apparatus

Steady-state fluorescence and excitation spectra were recorded at room temperature using an SLM-Aminco 8100 spectrophotometer equipped with a 450 W xenon lamp as a light source. UV‒VIS spectra were recorded on a Varian Cary 50 UV‒VIS spectrophotometer (Palo Alto, CA, USA). The baseline subtraction was applied to the spectra of dispersed particle to compensate the light scattering. FT-IR spectra were recorded using a Nicolet iS10 FTIR spectrometer (Thermo Fisher Scientific, Waltham, MA, USA) with an ATR accessory. SEM images were taken with a Phenom Pro operated at 5 or 10 kV equipped with a back scatter electron detector and a sample holder for charge reduction (Phenom World, Thermo Fisher Scientific, Waltham, MA, USA). Samples were sputtered with a 10 nm gold layer using a 208HR sputter coater (Cressington, Watford, UK). High-resolution transmission electron microscopy (HR-TEM) was performed using a Tecnai TF 20 X-TWIN microscope (FEI, Hilsboro, OR, USA) on samples dispersed with isopropanol on a copper grid. Centrifugation was carried out with an MPW-250 (MPW Med. Instruments, Warsaw, Poland). To obtain a well-dispersed suspension of particles, a vortex mixer and ultrasound bath (Polsonic Sonic 6, 480 W) were used. For solubilization of perylene and fluorescein, a 10 µl Hamilton syringe was used. Hydrodynamic diameters and dzeta potentials of SCAMS and SCMS were measured using dynamic light scattering (DLS) technique at 25 °C with a Zetasizer Nano Series instrument (Malvern Instruments, Malvern, UK) with a detection angle of 173°. For the measurements, the suspensions with the concentration of particles equal to 0.01 mg/ml were used. The textural parameters of the samples were determined by N_2_ sorption at − 196 °C using a 3Flex v.1.00 (Micromeritics) automated gas adsorption system. Prior to the analysis, the samples were degassed under vacuum at 90 °C for 24 h.

### Procedures

#### Synthesis of anthracen-9-ylmethyl(3-(triethoxysilyl)propyl)carbamate (TEOS-A)

9-(Hydroxymethyl)-anthracene (1.04 g, 4.99 mmol), 3-(triethoxysilyl)propyl-isocyanate (1.36 mL, 5.50 mmol) and dibutyltin dilaurate (0.3 mL, 0.51 mmol) were dissolved in 50 mL of DCM. The mixture was stirred at room temperature, and the reaction progress was controlled by TLC (SiO_2_: acetone/n-hexane, 1:4 v/v). After 4 days, the reaction was quenched with ca. 120 mL of saturated solution of Na_2_CO_3_. The organic layer was collected and washed with deionized water (2 × 100 mL). The solvent was evaporated, and the residue was partially purified by flash chromatography (SiO_2_: DCM/n-hexane, 4:1 v/v). Once most impurities were eluted, the product was washed off from the column with acetone. Then, the material was preabsorbed onto silica gel and finally purified by column chromatography (SiO_2_: acetone/n-hexane; 1:4 v/v) to obtain pure compound (0.91 g, 40%) as a light cream solid.

^1^H NMR (600 MHz, CDCl_3_) (Fig. S1) δ: 8.50 (s, 1H, ArH), 8.40 (d, 2H, ArH), 8.02 (d, 2H, ArH), 7.52 (m, 4H, ArH), 6.14 (s, 2H, CH_2_), 4.93 (br t, 1H, NH), 3.77 (q, 6H, OCH_2_), 3.22 (q, 2H, NCH_2_), 1.62 (quint, 2H, CH_2_), 1.17 (t, 9H, CH_3_), 0.65–0.58 (m, 2H, CH_2_Si);

MS (m/z): HRMS (ESI) (Fig. S2) Calcd. for C_25_H_33_NO_5_Si ([M + Na]^+^): 478.2020, found: 478.2018.

#### Synthesis of solid core anthracene-containing mesoporous shell (SCAMS) silica particles

Solid core mesoporous shell (SCMS) silica particles were synthesized following the recently reported procedure, which was modified here accordingly^9^. 120 ml of the suspension after the first stage of the synthesis (obtaining solid silica cores; here synthesized at 22 °C) was divided into six identical portions (6 × 20 ml). Afterwards, 40 mL of water and 8 mL of CTAB surfactant solution in ethanol/H_2_O (1:3 v/v) (m_CTAB_ = 0.239 g) were added to each portion of the dispersion of the solid cores, and the resulting mixtures were stirred for 15 min. In the next step, appropriate amounts of silica precursor (TEOS and TEOS-A) dissolved in ethanol (1 mL) were added, resulting in the molar ratios specified in Table 1. The mixtures were stirred overnight, and then the particles were centrifuged (5 min, 7900 RCF), washed with ethanol and dried at 50 °C overnight. The obtained white powders, SCAMS, labelled as shown in Table 1 (following the weight percentage of TEOS-A in the feed mixture), were stored in a desiccator in darkness.

**Table 1:** The quantities of silica precursors (TEOS and TEOS-A) used for the preparation of SCAMS.

	Sample	TEOS-A [mg]	TEOS [mL]	Molar ratio TEOS-A/TEOS	TEOS-A^a^ [µmol]
1	SCMS	–	0.43	–	–
2	SCAMS_1%	4	0.42	0.0046	8.8
3	SCAMS_3%	11.9	0.41	0.0141	26.2
4	SCAMS_5%	19.7	0.40	0.0240	43.3
5	SCAMS_10%	39.4	0.38	0.0504	86.6
6	SCAMS_20%	78.8	0.34	0.1127	173

^a^The total volume of each mixture was equal to 69.7 mL.

SCAMS particles were also subjected to the extraction procedure leading to SCAMS-EX. Briefly, 1 g of SCAMS was suspended in 250 mL of 0.37% HCl in EtOH. After 2 h of vigorous stirring, the material was centrifuged (5 min, 7900 RCF), and the extraction procedure was repeated once more. Finally, SCAMS-EX particles were isolated by centrifugation and dried at 50 °C. The final material was stored in a desiccator in darkness.

#### Förster resonance energy transfer (FRET) experiments

5 mL of Pe solution in THF (0.26 g/L) and 5 mL of Fl solution in PBS (0.378 g/L) were prepared in vials. PBS was used as a solvent for Fl in order to keep a constant pH since fluorescence of Fl is pH-dependent. SCAMS suspension was prepared by dispersing 10 mg of SCAMS in 10 ml of water (for experiments with Pe) or PBS (for experiments with Fl). All suspensions were sonicated for 15 min. Afterwards, 10 µL of Pe (or Fl) solution was added slowly to 2 mL of SCAMS suspension placed in a cuvette (quartz cuvettes with PTFE screw cap; 4 clear windows; 10 mm pathlength, Hellma, Germany) under vigorous stirring. All solutions and suspensions were prepared and used in the dark to avoid photodegradation.

#### Photosensitized oxidation of perylene encapsulated in SCAMS

10 µL of Pe solution (0.26 g/L in THF) was added slowly under vigorous stirring to 2 mL of SCAMS suspension in a quartz cuvette. Then, the sample was placed in a spectrofluorometer and irradiated using monochromatic light obtained in the apparatus (λ_irr_ = 320 nm). After each 10 min of irradiation, an emission spectrum of Pe (λ_ex_ = 410 nm) was recorded in the same instrument. Irradiation was performed for 80 min in total.

#### Separation and purification of SCAMS with solubilized perylene

Aqueous suspension of SCAMS (8 mL) with solubilized Pe, as obtained in the procedure 2.3.3 were subjected to centrifugation (5 min, 7900 RCF) and the supernatant was removed. Then water was added (8 mL) and the sample was shaken using a vortex for 5 min and then subjected to the same centrifugation, isolation and addition of water.

## Results and discussion

Anthracene-containing mesoporous silica particles (SCAMS) were synthesized using a common precursor, tetraethoxysilane (TEOS) and an appropriate An derivative of TEOS (TEOS-A) that was synthesized here. For the same synthetic conditions but various ratios of TEOS to TEOS-A, some differences in the yield were noticed. With increasing TEOS-A content, the yield slightly decreased, reaching ca. 87% of the weight of TEOS-only particles for the highest content of TEOS-A (20%) in the feed solution (SCAMS_20% particles) (Fig. S3). This can be rationalized by the presence of bulky An moieties in the modified silica precursor (TEOS-A), which may affect the rates of hydrolysis and condensation reactions proceeding during formation of the silica framework^37^. Morphologies and sizes of the obtained SCAMS particles were investigated by means of Scanning Electron Microscopy (SEM). SEM images of SCAMS_10% and SCMS (no An) indicated spherical morphology and low size dispersity of the particles disregarding the introduction of An (Fig. S4A and B). The diameters of SCAMS_10%, as an example, and SCMS particles were examined, and size histograms were plotted (Fig. S4C). The average diameter of SCAMS_10% particles (d_av_ = 512 nm) was found to be only ca. 6% lower than that of unmodified SCMS (d_av_ = 544 nm). This can be explained by favourable homo-condensation reactions of silanol groups compared to co-condensation with more bulky TEOS-A. It may lead to slightly less effective growth of the mesoporous shell in the case of SCAMS, but the overall effect is not significant. Comparable average hydrodynamic diameter was also found in DLS measurements of aqueous dispersion of SCAMS_5% while for SCMS slightly larger diameters were found (Fig. S5 and Table S1).

**Figure 1:**
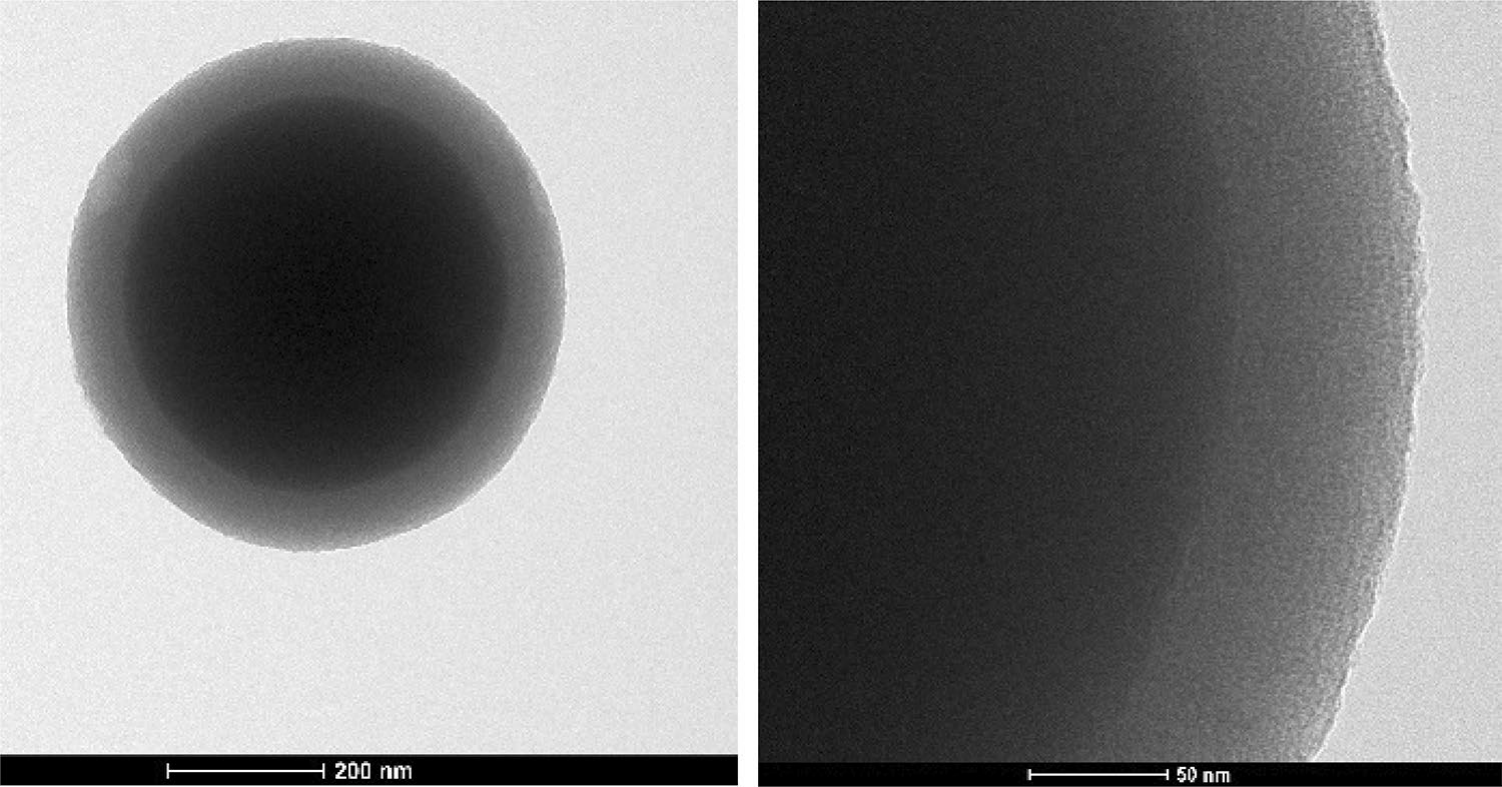
High-resolution TEM images of SCAMS_5%.

**Figure 2:**
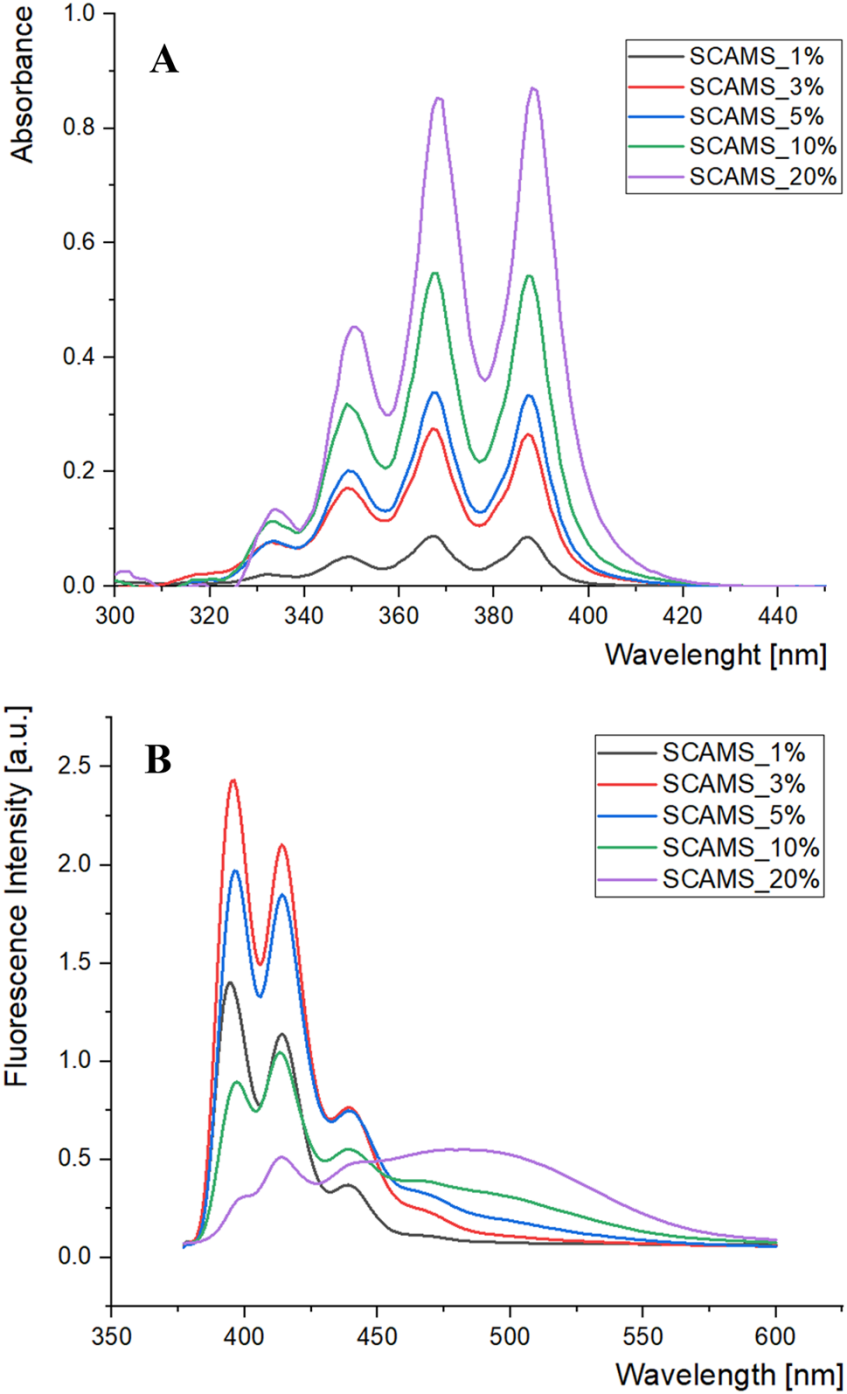
(**A**) UV‒Vis absorption spectra of as-synthesized SCAMS particles dispersed in THF (c = 1 mg/mL) and (**B**) the respective fluorescence spectra in aqueous solutions (c = 1 mg/mL; λ_ex_ = 330 nm).

A more detailed analysis of aged particles was performed using Transmission Electron Microscopy (TEM) to evaluate the stability of the mesoporous shell over time, which is crucial for their potential applications. TEM images of SCAMS_5% (Fig. 1) and the respective extracted SCAMS_5%(EX) (Fig. S6) were obtained 24 months after their synthesis to check the robustness of the mesoporous structures. Relatively mild (compare to calcination) ethanolic hydrochloric acid extraction methods was used here in order to preserve organic components it the formed mesoporous material. Such a method was previously showed to lead to complete removal of micellar template in similar systems^38^. In both cases, likely due to the presence of solid cores, the structure and spherical shape were preserved. However, the shell of the raw SCAMS_5% was found to be about twice as thick (approximately 80 nm) as that of the extracted SCAMS_5%(EX) (approximately 40 nm). The mesoporous structure of SCAMS_5%(EX) seemed to collapse or degrade during long storage, likely due to partial disintegration of the empty pore network, which was not the case for the pores filled with cylindrical micelles. The presence of TEOS-A in the reaction mixture might have introduced some disorder in pores compared to SCMS based exclusively on TEOS^39^, but mesopores may still be recognized in SCAMS even after 24 months of storage (Fig. 1). Importantly, the mesoporous character of the shell was confirmed using the adsorption/desorption isotherm measurement and density functional theory (DFT) model to determine pore size distribution. The surface area of SCAMS_5%(EX) was found to be 133 m^2^/g and two maxima around 1.5 nm and 2.8 were found in the distribution of the pore sizes (Fig. S7). UV‒VIS spectra of SCAMS particles dispersed in THF (Fig. 2A) indicated an increasing content of An in SCAMS with increasing concentration of TEOS-A in the feed mixture, followed by the characteristic absorption bands of An (330–400 nm). Importantly, SCAMS particles exhibited efficient fluorescence after dispersing in water (Fig. 2B) if compared to An that is only sparingly soluble in water. Upon excitation with λ_ex_ = 330 nm, the highest fluorescence intensity was observed for SCAMS_3% and only slightly lower for SCAMS_5%. In the case of materials with higher TEOS-A content, the fluorescence was quenched mainly due to the formation of excimers, which is indicated by the appearance of a characteristic broad band with a maximum above 450 nm^40^. In spite of a lower relative fluorescence intensity of SCAMS_5% than SCAMS_3%, the former one exhibited more efficient excitation energy transfer to encapsulated energy acceptor (Fig. S8; see later for detailed discussion on energy transfer) and SCAMS_5% particles were selected for further photochemical studies.

To verify whether An groups were covalently attached to the silica network, SCAMS particles were subjected to acidic extraction. As a result, particles without cylindrical micelle templates were obtained (SCAMS-EX)), and their fluorescence spectra were measured (Fig. S9). A typical treatment of SCAMS with a solution of hydrochloric acid in ethanol resulted in the removal of surfactants but also potentially unbonded TEOS-A. In addition, the overall fluorescence of SCAMS-EX was weaker than that of SCAMS and the SCAMS-EX emission intensity increased linearly with increasing TEOS-A content in the feed mixture without changes in the spectral profiles. The results indicate that incorporation of An chromophores into the silica structure was nearly proportional to the initial concentration of the TEOS-A precursor. The composition of SCMS as well as SCAMS before and after extraction of the micellar template were also compared using FTIR spectroscopy (Fig. S10 and detailed discussion in SI). The content of TEOS-A may be evidenced in SCAMS_5% by the presence of bands at around 1670 cm^−1^ (C=O stretching), and 1594 cm^−1^ (N–H bending) that can be assigned to the groups present in TEOS-A. After extraction, the content of those groups is smaller, likely due to removal of unbound TEOS-A, so the bands diminish. While partial detachment of the bonded TEOS-A groups cannot be excluded even in the mild extraction condition we chose, FTIR spectrum of SCAMS_5%-EX shows some remaining C–H stretching bands (below 3000 cm^−1^) that cannot be assigned to residual CTAB (2850 cm^−1^ and 2920 cm^−1^) thus indicating the presence of bonded organic silane (TEOS-A).

**Figure 3:**
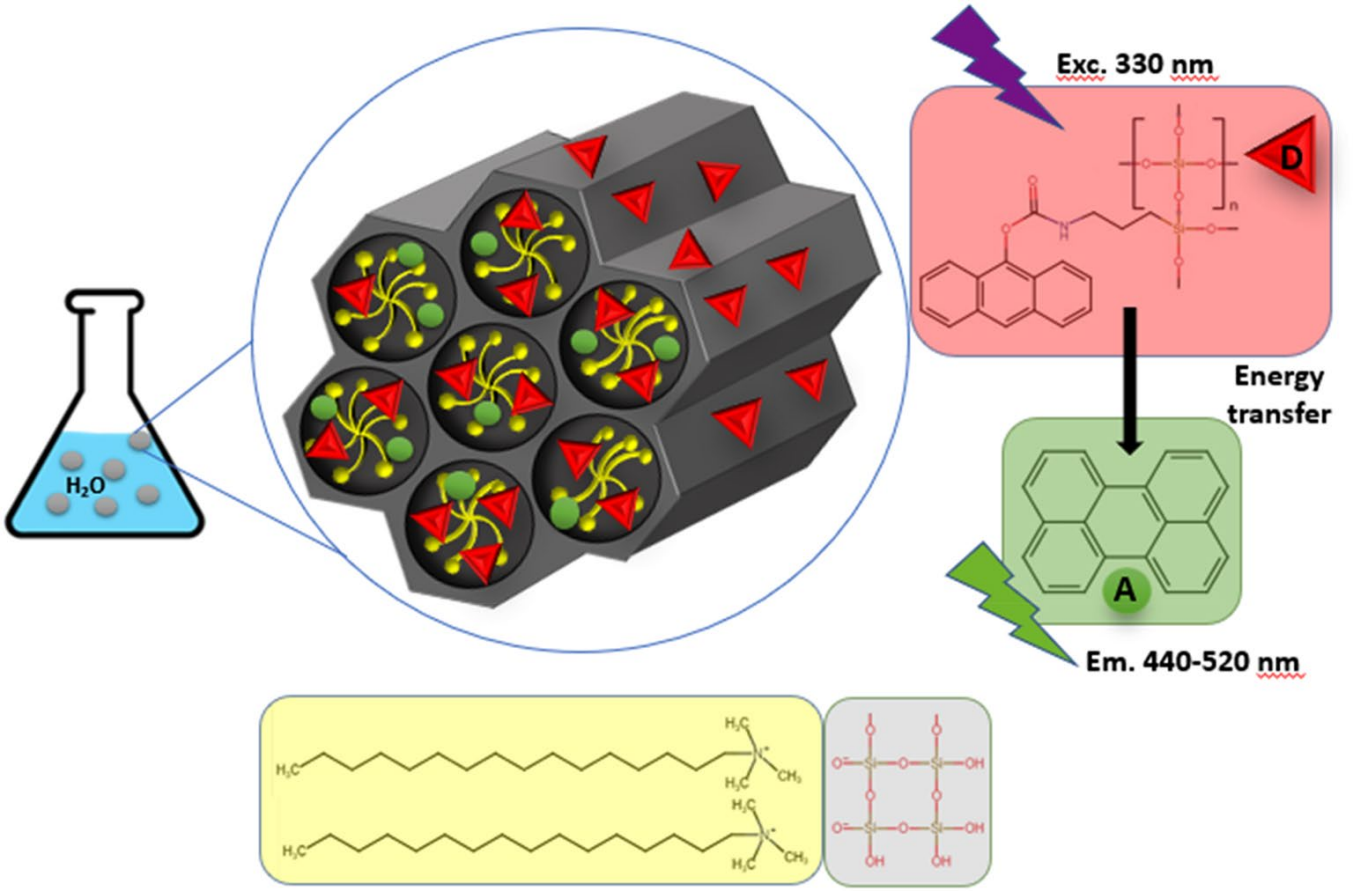
Scheme of the SCAMS system used for realization of FRET in the mesopores between An (donor) chromophores and encapsulated Pe (acceptor).

Such An-embedded SCAMS particles dispersed in water were then tested as carriers of hydrophobic compounds that may also take part in excitation energy transfer, with An chromophores serving as energy donors (Fig. 3).

**Figure 4:**
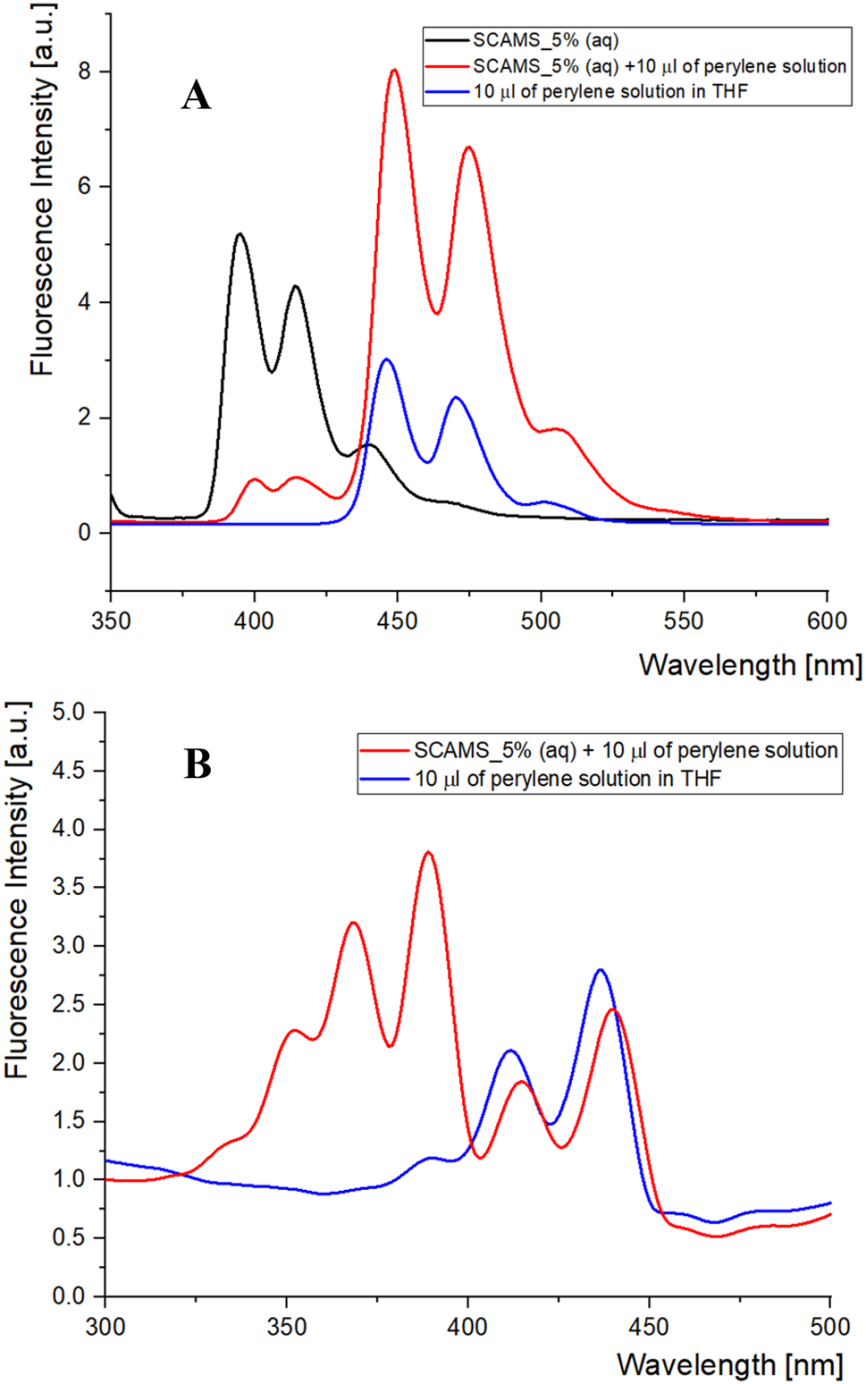
(**A**) Fluorescence spectra of SCAMS_5% with and without solubilized Pe dispersed in water (c = 1 mg/mL) as well as Pe solution in THF with the same total concentration (λ_ex_ = 330 nm); (**B**) excitation spectra of the same SCAMS_5% with solubilized Pe and Pe solution in THF (λ_em_ = 520 nm).

First, a small portion of a hydrophobic fluorophore, Pe, was solubilized within the mesoporous shell of SCAMS. Importantly, solubilization of Pe in SCAMS-EX was not effective due to the lack of hydrophobic domains in the mesopores. Pe was selected as a model energy acceptor for the FRET process since the An emission spectrum significantly overlaps the Pe absorption spectrum, leading to a reasonably high Förster critical radius (R_0_). The value of R_0_ for An-Pe pair (R_0_ ≈ 6 nm) was calculated based on the emission spectra of An and absorption spectra of Pe and Fl available in PhotochemCAD^41^. FRET was then monitored using fluorescence measurements. The fluorescence spectrum of An (λ_ex_ = 330 nm) present in SCAMS was found to decrease significantly in the same system after the addition of Pe undergoing solubilization in the rod-like micelles within the mesopores (Fig. 4A). The simultaneous increase of the acceptor (Pe) emission is an indication of efficient FRET between An and Pe in such a confined environment. Based on those spectra also the efficiency of FRET (Φ_FRET_ = 0.82) and the mean D-A distance (R = 4.7 nm) were estimated (see SI for details). The control experiments with Pe solution in THF (the same concentration as its total concentration in SCAMS_5%) and the same excitation wavelength (330 nm) that is preferentially absorbed by An, resulted in much smaller Pe emission than the one observed by indirect excitation via An. This may also be supported by the measured excitation spectra at the wavelength characteristic of Pe emission only (520 nm) (Fig. 4B). For the SCAMS system, the contribution of the indirect An excitation (bands between 325 and 400 nm) is much larger than the direct excitation of Pe (bands between 375 and 450 nm). Thus, the observed effect is also not related to possible trivial radiative transfer. Additionally, a control experiment with TEOS-A solution in THF was performed (Fig. S11A). The absorbance of TEOS-A was kept the same as that of SCAMS_5% (see Fig. S12), but after the addition of the same amount of Pe, there was only a negligible increase in the acceptor emission after excitation of the donor (λ_ex_ = 330 nm). Such a small increase may also be related to the direct excitation of Pe at that wavelength (Fig. S11B). Thus, no FRET was observed, indicating that the large average distance between the donor and acceptor molecules in solution and efficient FRET should be related to the confinement effect created in the mesoporous shell of SCAMS. Similarly, only negligible FRET (Fig. S13) was noticed if Pe was attempted to be solubilized in the extracted SCAMS using the same condition as for raw SCAMS and even the particles with the highest An content were used (SCAMS_20%-EX). It can be explained by the lack of sufficiently hydrophobic domains that would keep Pe inside the mesopores. SCAMS_5% with solubilized Pe were also isolated and purified (see procedure 2.3.5). The shape of the spectra did not change for such purified and redispersed particles indicating efficient solubilization of Pe in the pores but not only in the potentially released to solution micellar templates (Fig. S14). The observed decrease of the overall fluorescence intensity after separation and purification of the particles may be related to the loss of some particles during the procedure, their partial disintegrations during strong centrifugation as well discarding of some micellar templates released from the particles.

**Figure 5:**
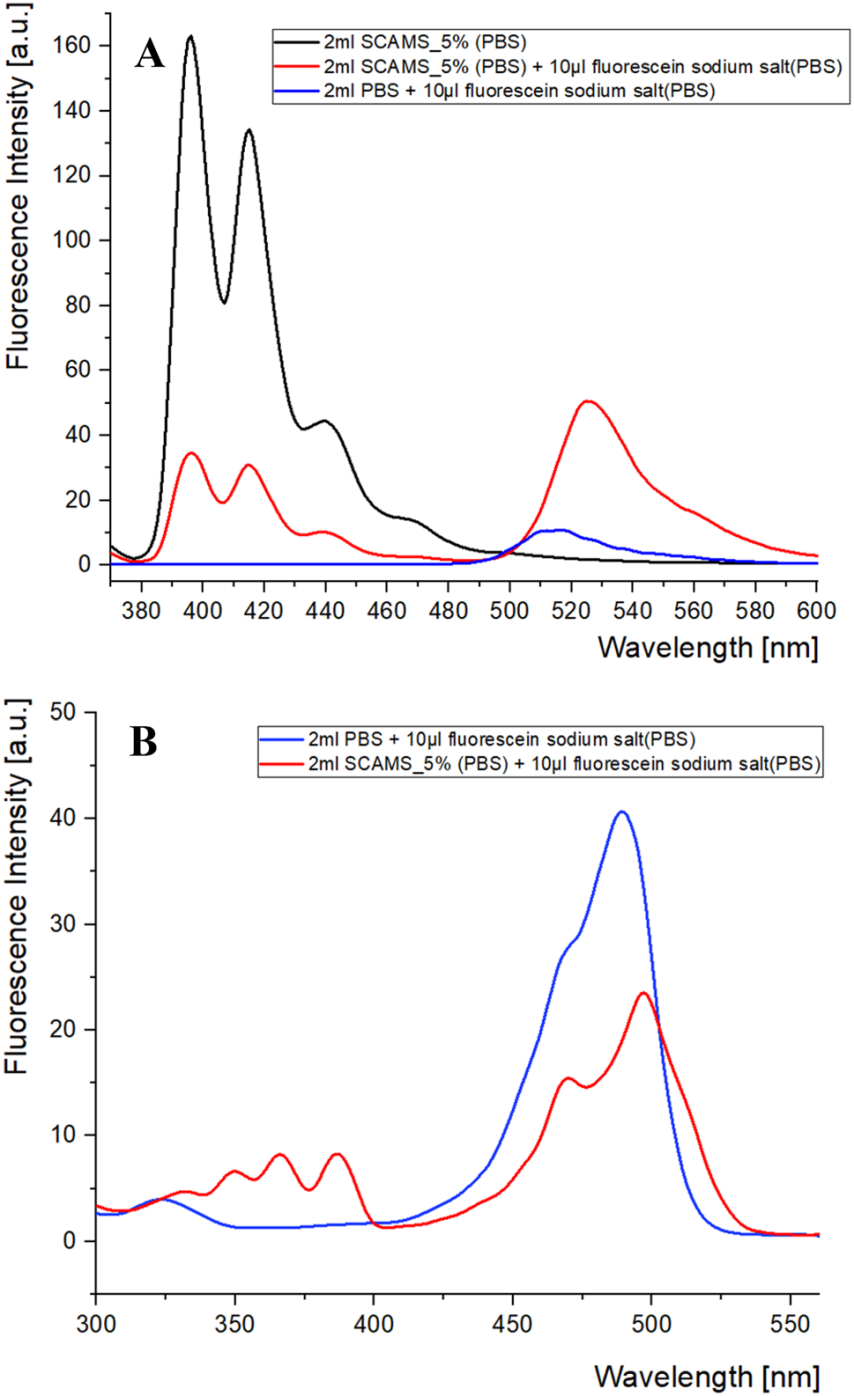
(**A**) Fluorescence spectra of SCAMS_5% dispersed in water (c = 1 mg/mL) before and after the addition of Fl in PBS solution (λ_ex_ = 350 nm). (**B**) Excitation spectra of the same system (λ_em_ = 580 nm).

Furthermore, we wanted to check whether a similar effect could be observed for SCAMS using a water-soluble sodium salt of fluorescein (Fl) as an energy acceptor. Surprisingly, efficient FRET was also observed for such a system, indicating close proximity of the donor and acceptor molecules within the pores (Fig. 4A,B). The Förster critical radii for An-Fl (R_0_ ≈ 6 nm) donor–acceptor pair was determined similarly as for An-Pe pair. Based on the respective spectra (Fig. 5A) also the efficiency of FRET (Φ_FRET_ = 0.78) and the mean D-A distance (R = 5.6 nm) were estimated for An-Fl pair (see SI for details). The occurrence of FRET may also be supported by the measured excitation spectra at the wavelength characteristic of Pe emission only (580 nm) (Fig. 5B). Thus, the obtained results indicate that Fl molecules are also able to penetrate the mesopores of SCAMS, likely occupying the interface between the silica pores and the cylindrical micelles. If Fl molecules would be only available at the surface of SCAMS, such efficient FRET would be unlikely due to too large a distance from the embedded An chromophores.

**Figure 6:**
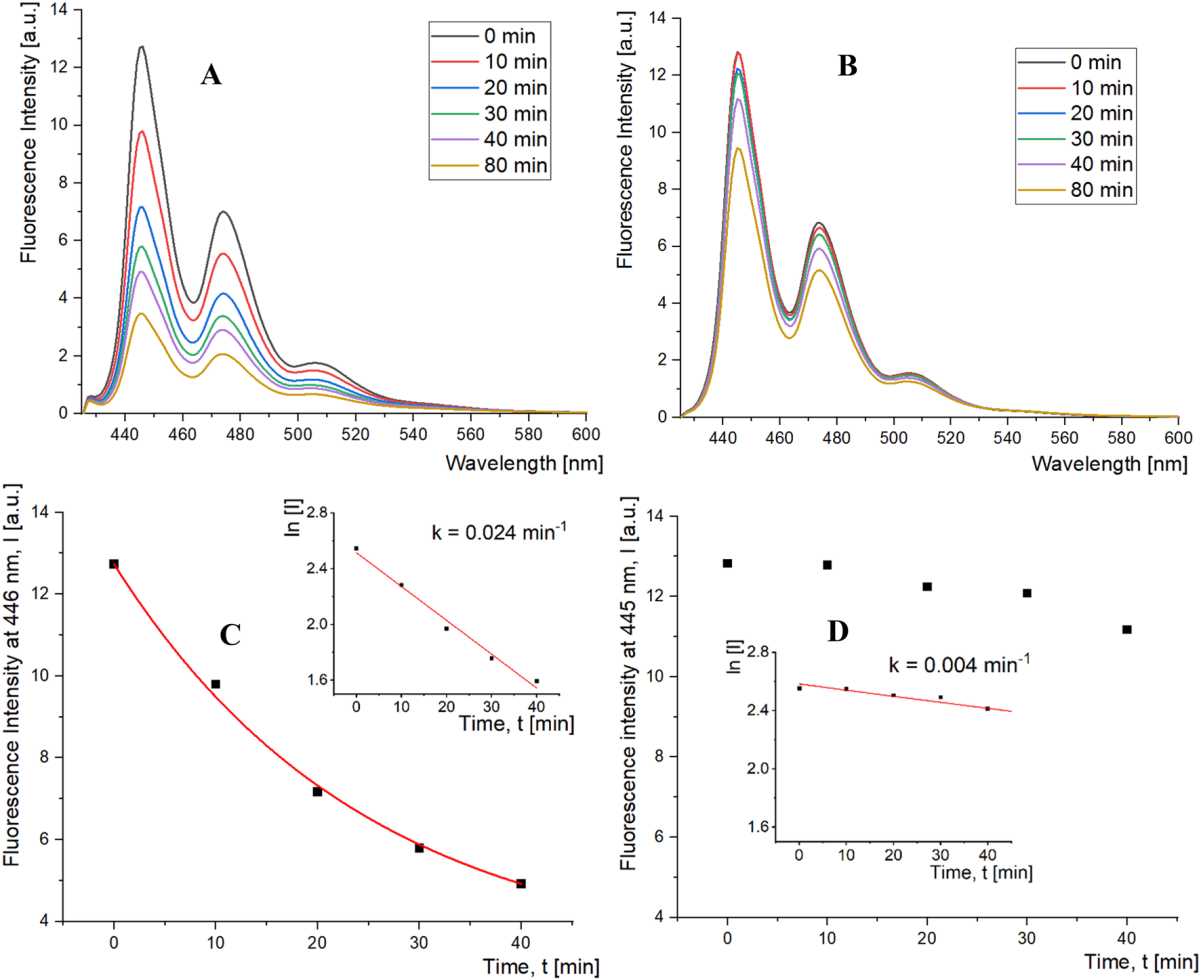
Fluorescence spectra of perylene (λ_ex_ = 410 nm) solubilized in: (**A**) SCAMS_5%, (**B**) SCMS; (particles were dispersed in water, 1 mg/mL) after various irradiation times (λ_irr_ = 320 nm). Decays of Pe fluorescence measured at the maximum emission wavelength for the (**C**) SCAMS_5% and (**D**) SCMS systems after various irradiation times. First-order kinetic plots (ln(I) = f(t)) together with the respective rate constants are shown in the insets.

Finally, we performed photosensitized oxidation of Pe using an irradiation wavelength (λ_irr_ = 320 nm) absorbed almost exclusively by An chromophores present in SCAMS particles but insignificantly absorbed by Pe. Figure 6A shows the spectra of Pe emission in the SCAMS/Pe system after various irradiation times, illustrating the process of photooxidation of Pe to nonfluorescing perylenequinones^29^. The decrease in fluorescence intensity unambiguously indicates the progress of the photosensitized degradation of Pe in SCAMS. The first-order kinetics was applied to determine the rate constant of this process, which was found to be six times faster than that performed in a control SCMS system without An chromophores (Fig. 6B–D), pointing to efficient photosensitization in such a confined space. Additional oxygenation of the systems did not bring any significant increase in the oxidation rates of Pe, indicating that the concentration of oxygen in both systems was not the rate-limiting factor.

## Conclusions

In conclusion, solid-core silica particles with mesoporous shells containing immobilized anthracene chromophores (SCAMS) and mesopores filled with cylindrical micelles were shown to serve as water-dispersable microphotoreactors. The particles had submicrometer dimeters that are appropriate for their efficient isolation from aqueous medium using simple centrifugation, which might be damaging or inefficient for purely mesoporous nanoparticles. Due to the presence of cylindrical micelles unremoved from the mesopores, a confined hydrophobic environment was provided that can host hydrophobic molecules, enabling close proximity of the energy donor (An) and solubilized acceptor molecules. In such particles dispersed in water, efficient FRET from excited An to solubilized perylene and subsequent photosensitized oxidation was observed. Importantly, FRET was not observed for SCAMS-EX particles with the template micelles removed from the mesoporous structure, pointing to a vital role of the immobilized micelles in the performance of such novel photoreactors. Surprisingly, hydrophilic molecules (fluorescein, Fl) can also be placed in the nanophase created within the mesopores, as evidenced by the observation of FRET from the excited An to Fl. Due to the solid core mesoporous shell structure of the particles, they exhibited long-term stability and can be easily handled (centrifugation, sonication) in an aqueous dispersion that would not be possible for only micellar (susceptible to dilution) or entirely mesoporous particles (fragile). Further studies are supposed to address the versatility of SCAMS as a microphotoreactor for the efficient collection and photodegradation of hydrophobic but also hydrophilic impurities of water.

### Supplementary Information

Supplementary Information.

## Data Availability

Data is provided within the manuscript or supplementary information files.
