# Author Correction: Water-dispersable photoreactors based on core–shell mesoporous silica particles

**DOI:** 10.1038/s41598-024-64035-2

**Published:** 2024-06-05

**Authors:** Andrzej Baliś, Dominika Lorens, Arkadiusz Gut, Szczepan Zapotoczny

**Affiliations:** 1 Faculty of Chemistry, Jagiellonian University, Gronostajowa 2, 30-387 Krakow, Poland; 2 Jerzy Haber Institute of Catalysis and Surface Chemistry, Polish Academy of Sciences, Niezapominajek 8, 30-239 Krakow, Poland

Correction to: *Scientific Reports* 10.1038/s41598-024-61750-8, published online 17 May 2024

The Acknowledgments section in the original version of this Article was incomplete.

“Dr. Tomasz Uchacz is acknowledged for his help in calculations. Dr Joanna Odrobińska-Baliś is acknowledged for her help in preparing graphics.”

now reads:

 “Dr. Tomasz Uchacz is acknowledged for his help in calculations. Dr Joanna Odrobińska-Baliś is acknowledged for her help in preparing graphics. The open-access publication of this article has been supported by a grant from the Faculty of Chemistry under the Strategic Programme Excellence Initiative at Jagiellonian University.”

The original Article has been corrected.

