# Supplementary material for: Water-dispersable photoreactors based on core–shell mesoporous silica particles
Source: Sci Rep. 2024 May 17;14:11257. doi: 10.1038/s41598-024-61750-8 (PMC11099024; doi:10.1038/s41598-024-61750-8)
Supplement: Supplementary file 1 — Supplementary Information. [file 41598_2024_61750_MOESM1_ESM.pdf]

Supplementary Information

Water-dispersable photoreactors based on core-shell mesoporous silica particles

Andrzej Baliś,[1,2] Dominika Lorens,[1] Arkadiusz Gut,[1] Szczepan Zapotoczny[1,*]

[1] Jagiellonian University, Faculty of Chemistry, Gronostajowa 2, 30-387 Krakow, Poland

[2] Jerzy Haber Institute of Catalysis and Surface Chemistry, Polish Academy of Sciences, Niezapominajek 8, 30-239 Krakow, Poland

Supplementary Figures

δ_H (300 MHz, $CDCl_3$) 8.50 (1 H, s), 8.40 (2 H, d, J = 8.9 Hz), 8.02 (2 H, d, J = 7.9 Hz), 7.57 (2 H, t, J = 8.0 Hz), 7.48 (2 H, t, J = 8.2 Hz), 6.14 (2 H, s), 4.92 (1 H, br t), 3.77 (6 H, q, J = 7.1 Hz), 3.29 – 3.12 (2 H, m), 1.70 – 1.53 (2 H, m), 1.17 (9 H, t, J = 7.0 Hz), 0.66 – 0.55 (2 H, m).

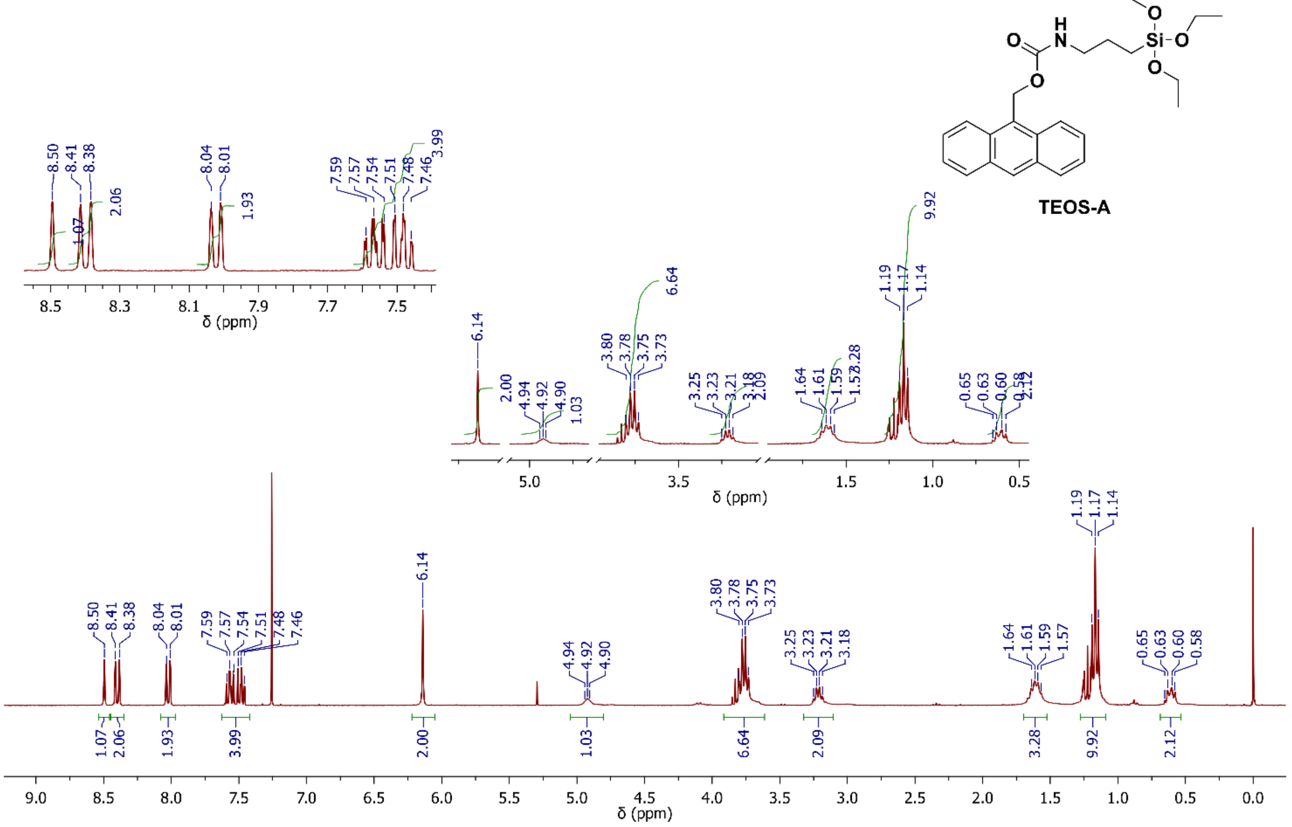

Figure S1. ^{1}H NMR spectrum of **TEOS-A** recorded in $CDCl_3$.

Acquisition Parameter

Source Type	ESI	Ion Polarity	Positive	Set Nebulizer	0.4 Bar
Focus	Not active			Set Dry Heater	180 °C
Scan Begin	50 m/z	Set Capillary	4500 V	Set Dry Gas	4.0 l/min
Scan End	3000 m/z	Set End Plate Offset	-500 V	Set Divert Valve	Waste

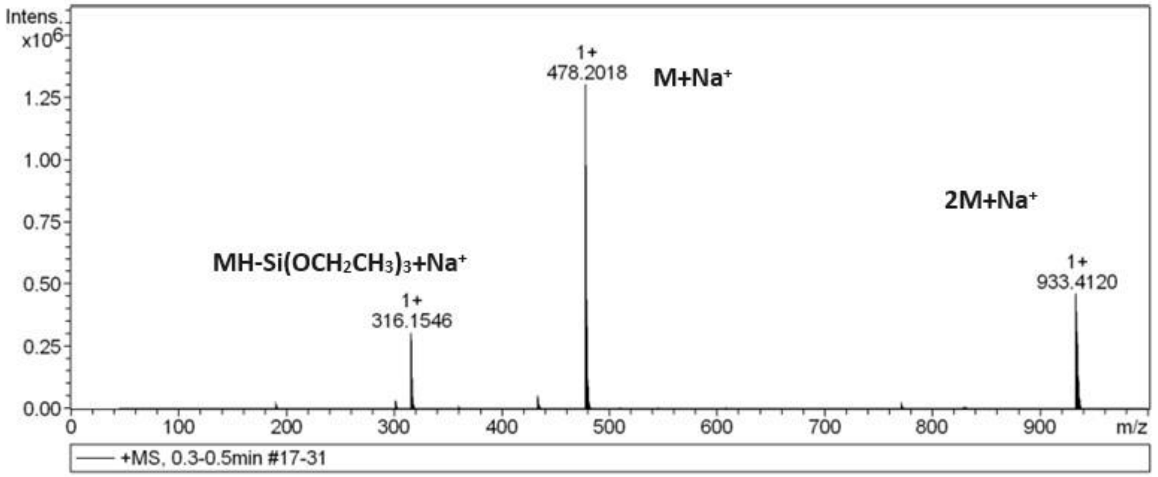

#	m/z	Res.	S/N	I	FWHM
1	191.0847	6563	1208.4	15932	0.0291
2	302.1385	7786	1002.3	33179	0.0388
3	303.1409	7799	181.4	6043	0.0389
4	316.1546	7680	8998.2	307642	0.0412
5	317.1567	7532	1618.9	55356	0.0421
6	318.1541	7606	511.4	17511	0.0418
7	360.3221	8137	258.0	9831	0.0443
8	434.2113	8633	1252.7	53502	0.0503
9	435.2137	8545	400.5	17098	0.0509
10	436.2138	8521	112.3	4792	0.0512
11	478.2018	7921	29161.9	1300253	0.0604
12	478.7599	2457	86.6	3860	0.1949
13	479.2045	8194	9907.2	441404	0.0585
14	480.2038	8386	2748.2	122325	0.0573
15	481.2048	8689	513.8	22865	0.0554
16	510.1908	8957	98.3	4376	0.0570
17	546.1880	9063	89.4	3948	0.0603
18	609.3196	9573	170.0	7149	0.0637
19	771.3656	10187	322.3	14133	0.0757
20	772.3683	9944	172.2	7556	0.0777
21	829.5131	10008	97.6	4213	0.0829
22	831.5249	10257	95.3	4105	0.0811
23	933.4120	9727	11814.2	462580	0.0960
24	934.4149	9792	7923.5	310084	0.0954
25	935.4149	9922	3428.8	134111	0.0943

Figure S2. HRMS spectrum of **TEOS-A** (positive ionization mode).

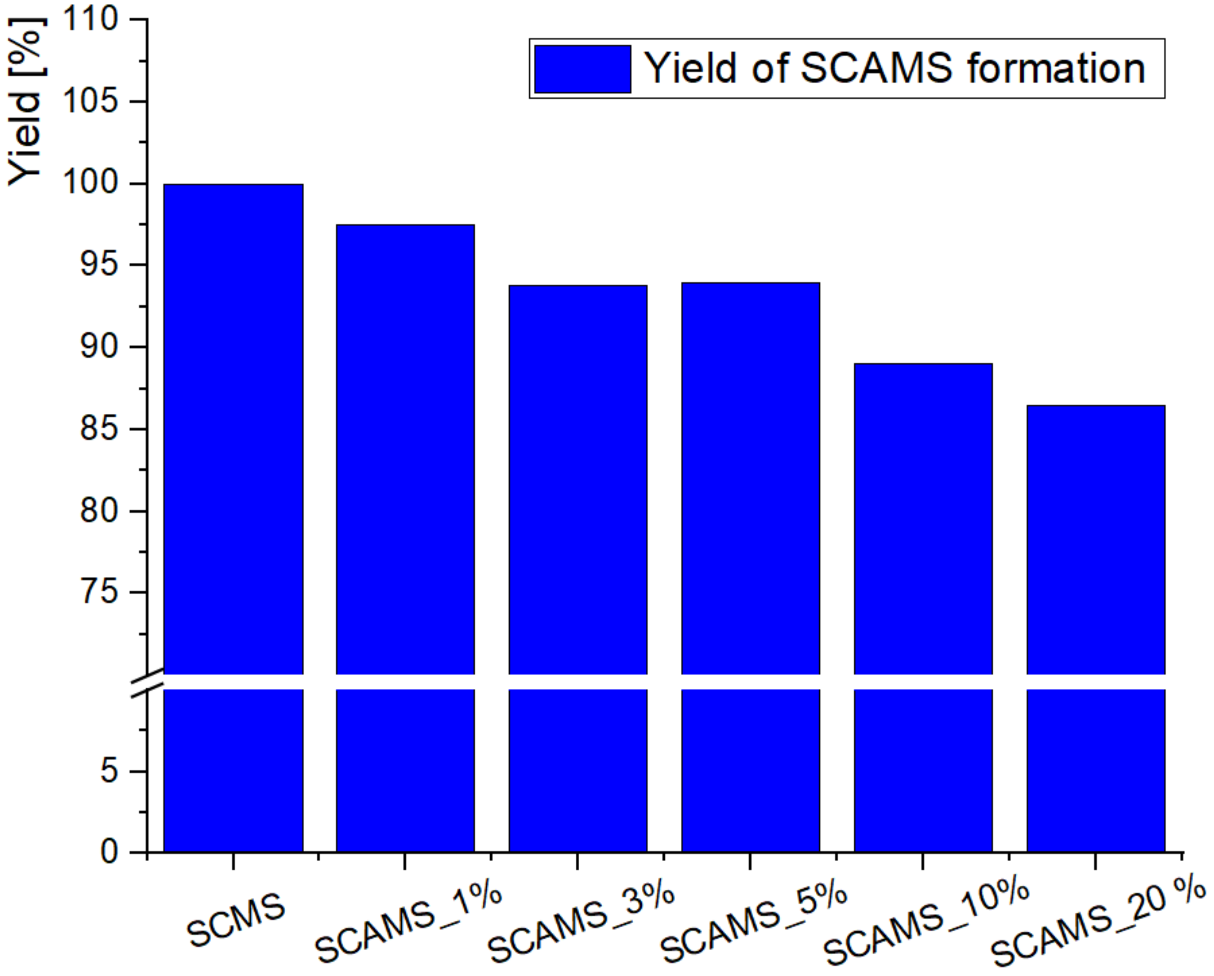

Figure S3. Relative yields of syntheses of SCAMS with respect to the yield of synthesis of SCMS particles.

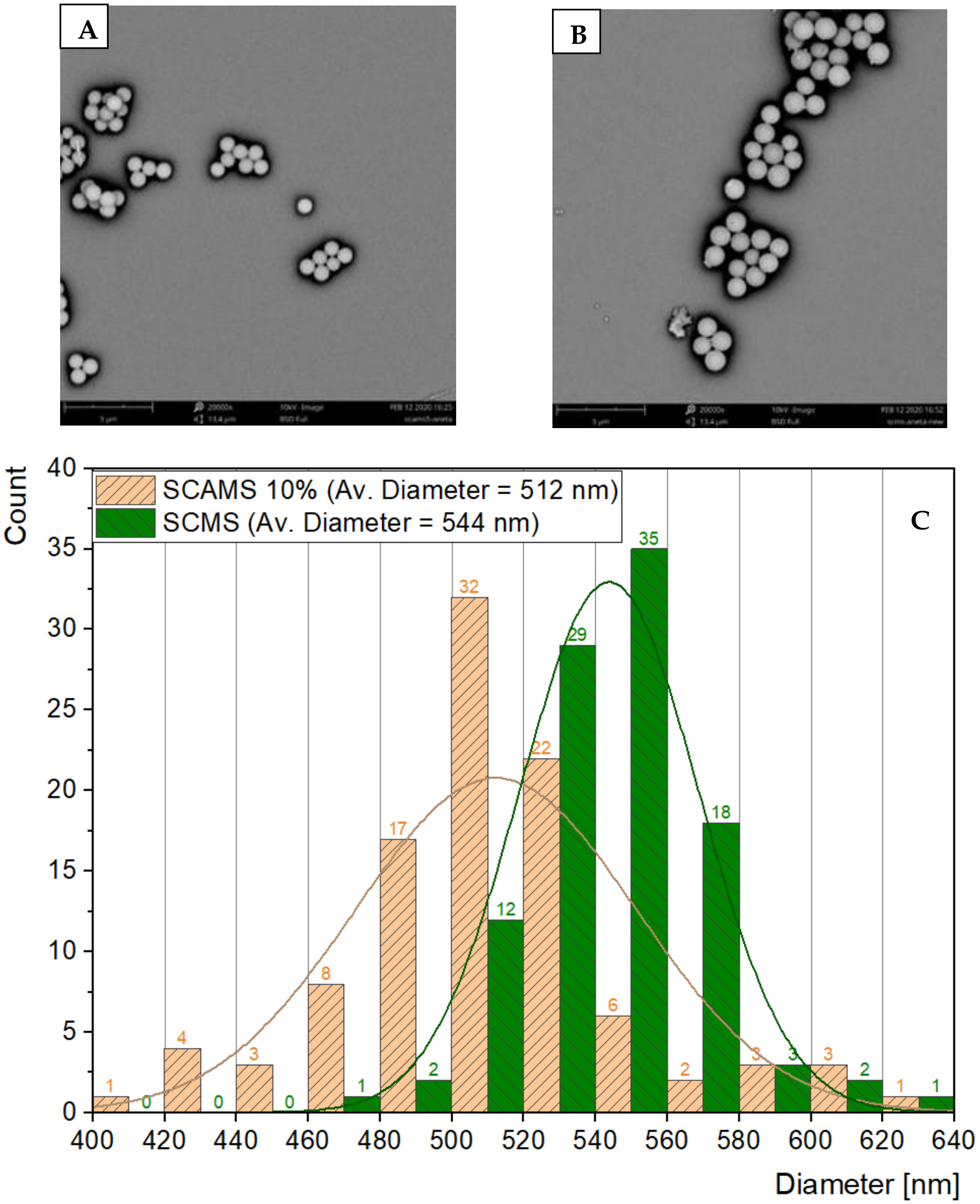

Figure S4. Scanning Electron Microscope images of: SCAMS 10% (A), SCMS (B) and (C) histogram based on the SEM images showing distribution of diameters of both types of particles.

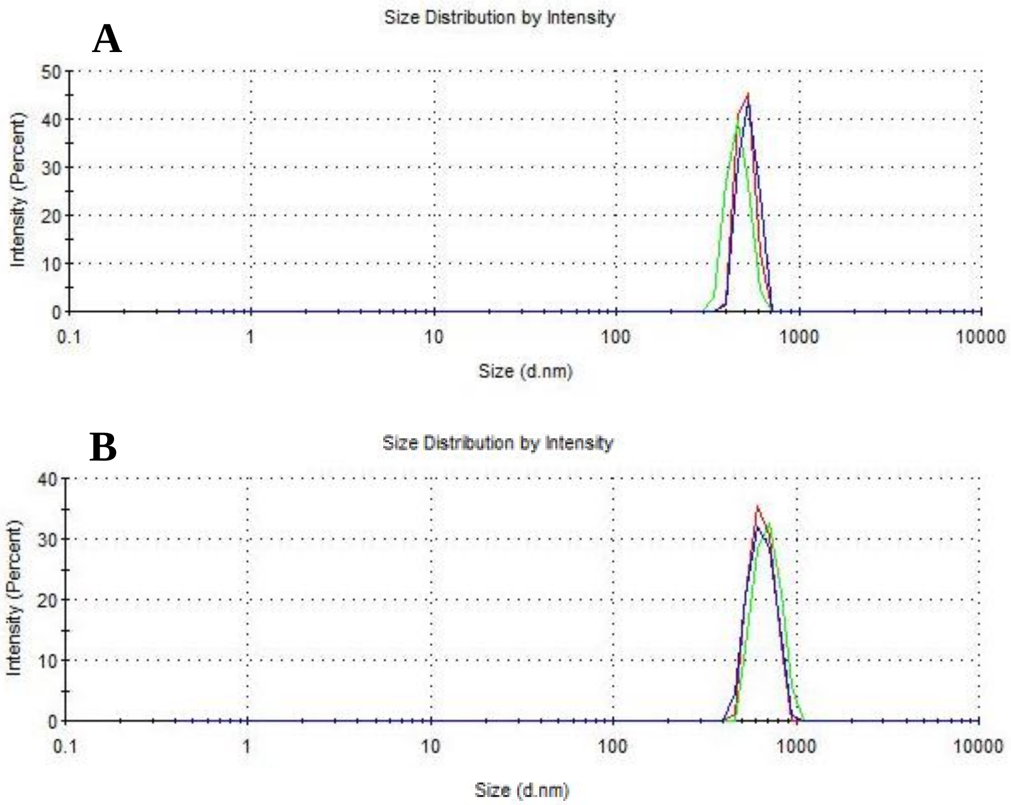

Figure S5. Example DLS results for aqueous dispersions of A: SCAMS_5% and B: SCMS

Table S1. Average hydrodynamic diameters, polydispersity indexes (PDI) and zeta potential values SCMS and SCAMS_5% dispersed in water (based on the data presented in Figure S5).

Sample	Average diameter (by intensity) [nm]	Average diameter (by volume) [nm]	Average diameter (by number) [nm]	PDI	Zeta potential [mV]
**SCMS (AV)**	**646**	**668**	**693**	**0.16**	**+32.8**
**SCAMS_5% (AV)**	**492**	**501**	**513**	**0.45**	**+57.4**

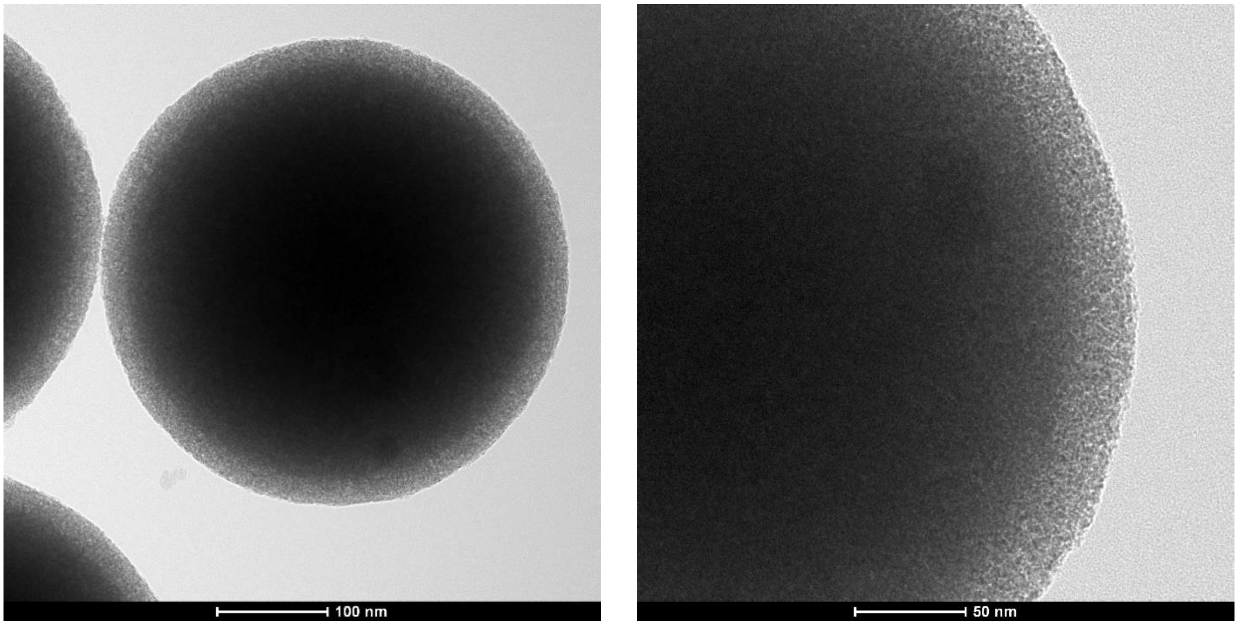

Figure S6. HR-TEM images of SCAMS_5%(EX).

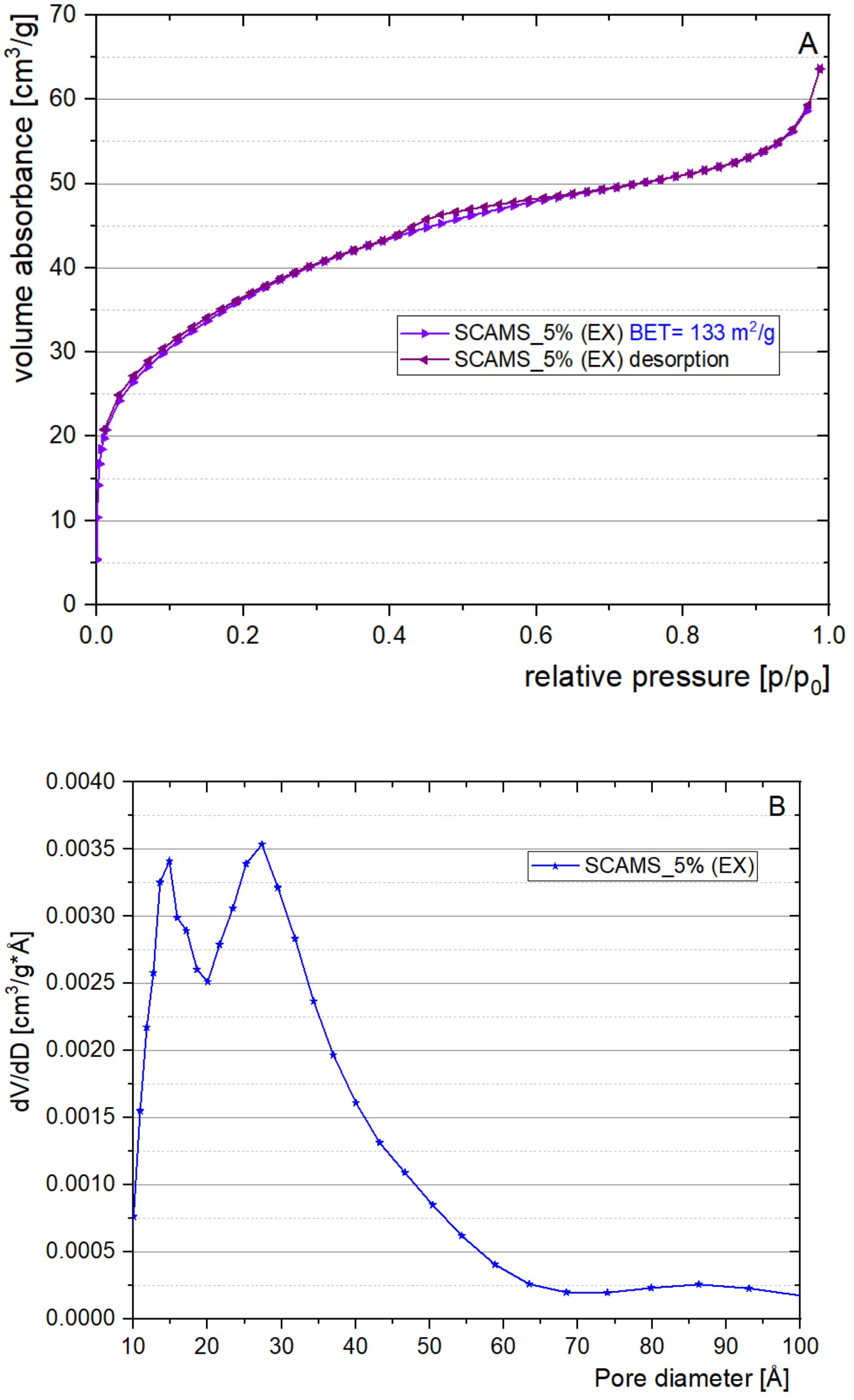

Figure S7. Adsorption-desorption plot and BET isotherm for SCAMS_5%(EX) after 2 years of storage and the corresponding porosity distribution by original Density Functional Theory (DFT) model (B).

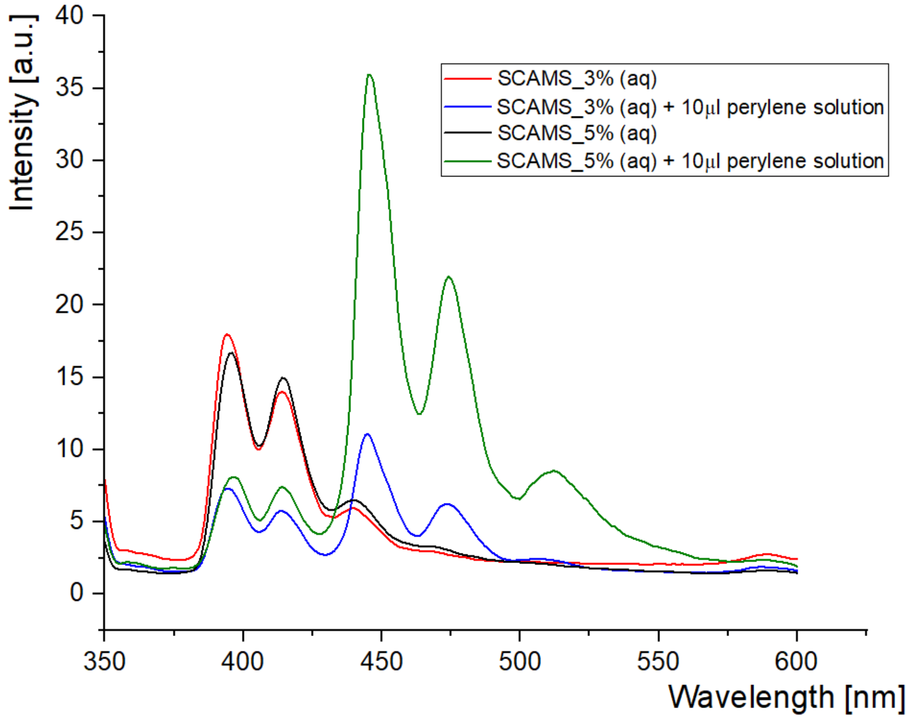

Figure S8. Fluorescence spectra of SCAMS_5% and SCAMS_3% dispersed in water without and with solubilized Pe (λ_{ex} = 330 nm).

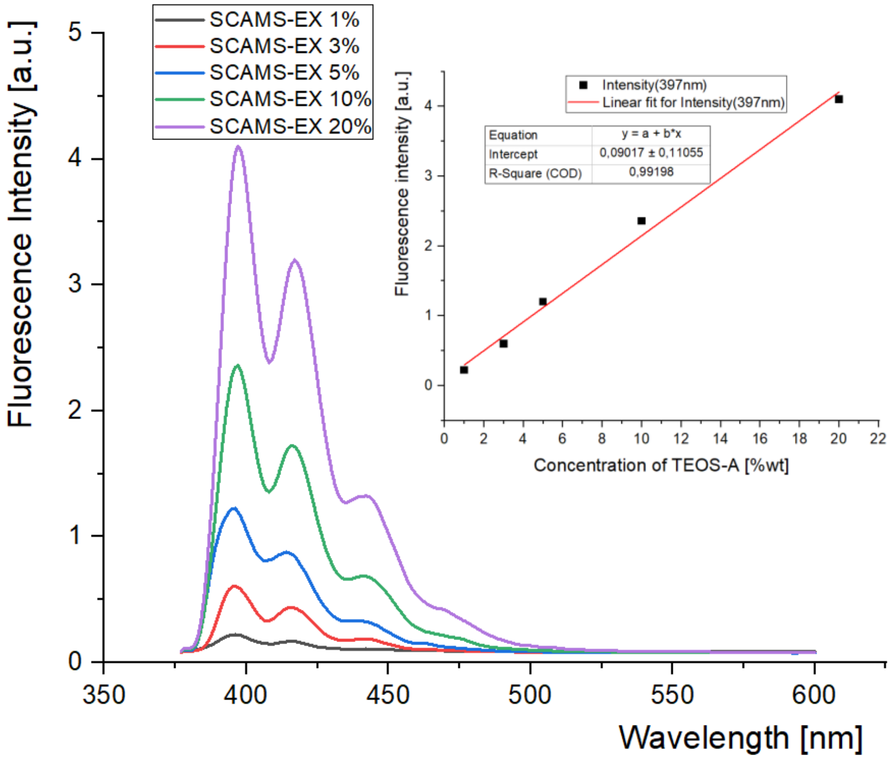

Figure S9. Fluorescence spectra of SCAMS-EX particles (λ_{ex}= 330 nm).

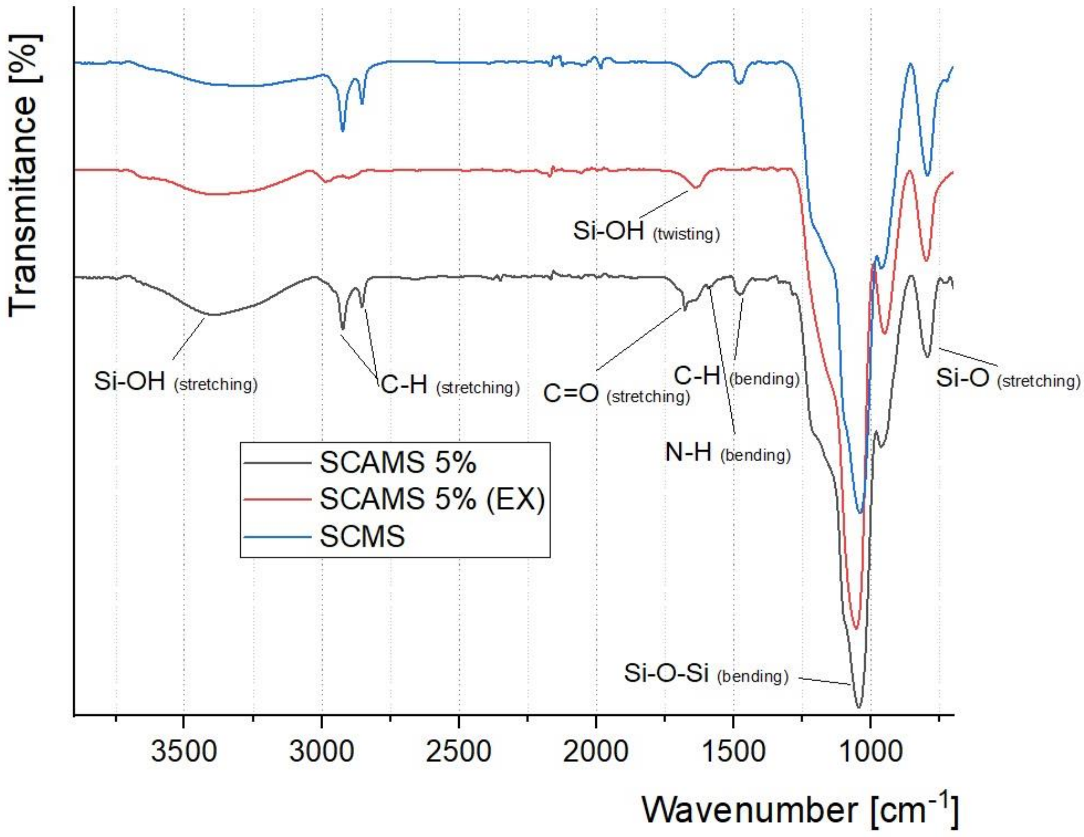

Figure S10. FTIR spectra of SCMS, SCAMS_5%, and SCAMS_5%(EX) particles.

As shown in the presented FTIR spectra (Figure S10) CTAB is practically completely removed after the extraction procedure – the C-H bending band (1470 cm^{-1}) and sharp C-H stretching bands (2850 cm^{-1} and 1920 cm^{-1}) disappeared.[1] Some residual C-H stretching bands, shifted with respect to intense bands assigned to CTAB, may be assigned to the groups present in the bonded TEOS-A organosilane and some residual partially hydrolyzed TEOS molecules.[2] The content of TEOS-A may be evidenced in SCAMS_5% by the presence of bands at around 1670 cm^{-1} (C=O stretching), and 1594 cm^{-1} (N-H bending) that can be assigned to the groups present in TEOS-A only. After extraction, the content of those groups is smaller (removal of unbound TEOS-A, detachment of the bounded TEOS-A) so the bands diminish.

A

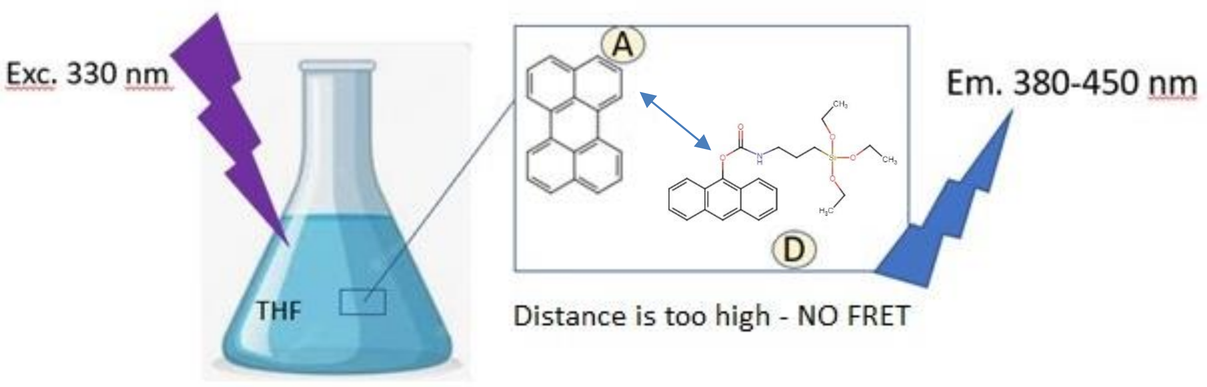

B

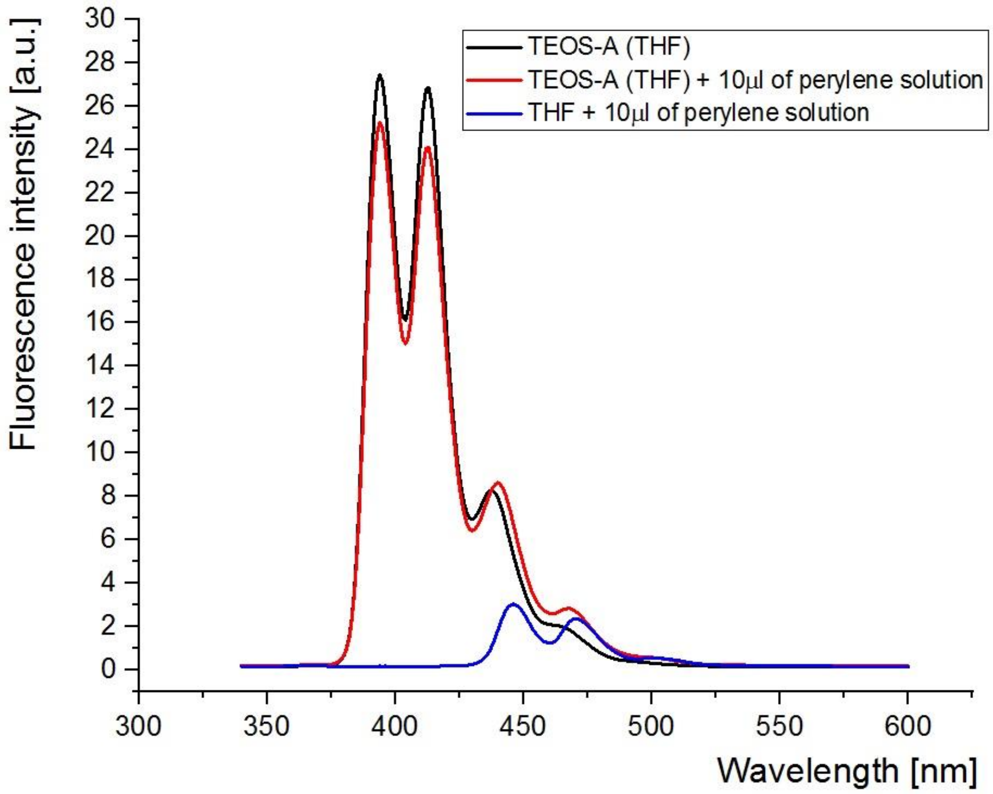

Figure S11. Scheme of a control experiment (A): excitation of TEOS-A in a THF solution with addition of Pe and the respective emission spectra (B) of TEOS-A before and after addition of Pe, as well as the solution with the same concentration of Pe (λ_{ex} = 330 nm).

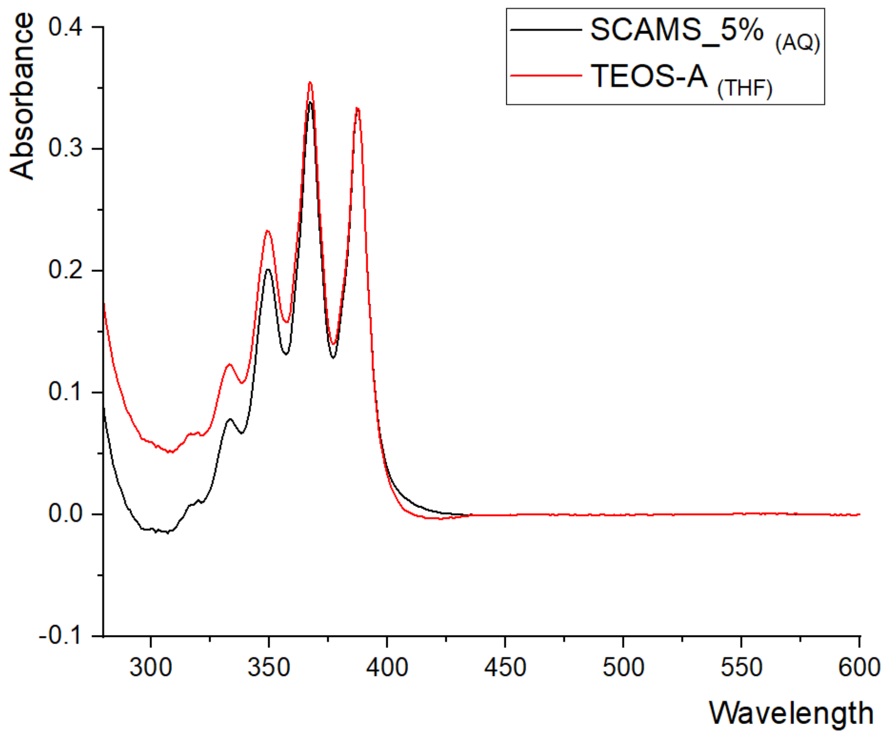

Figure S12. UV-Vis absorption spectra of SCAMS_5% dispersed in water and TEOS-A in THF solutions at concentrations used in comparative experiments on energy transfer to Pe.

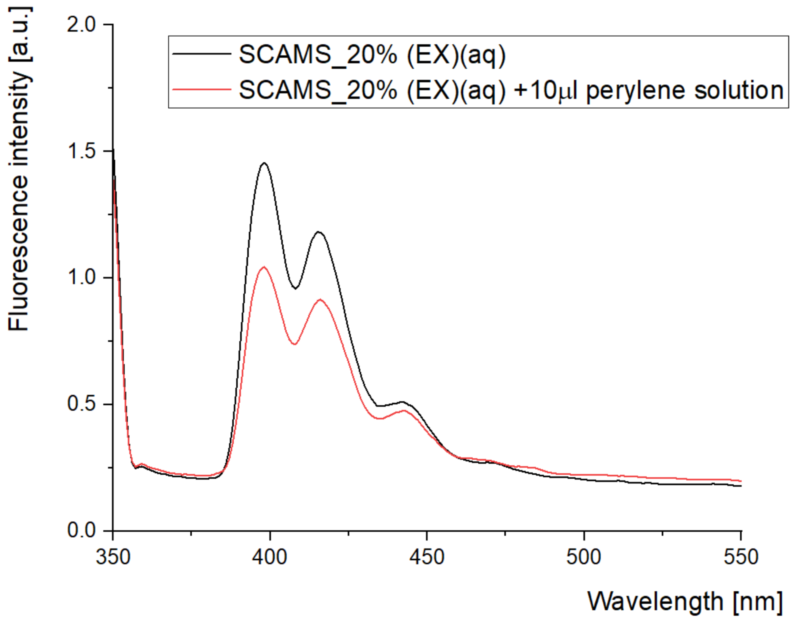

Figure S13. Fluorescence spectra of SCAMS_20%-EX without and with solubilized Pe (λ_{ex} = 330 nm).

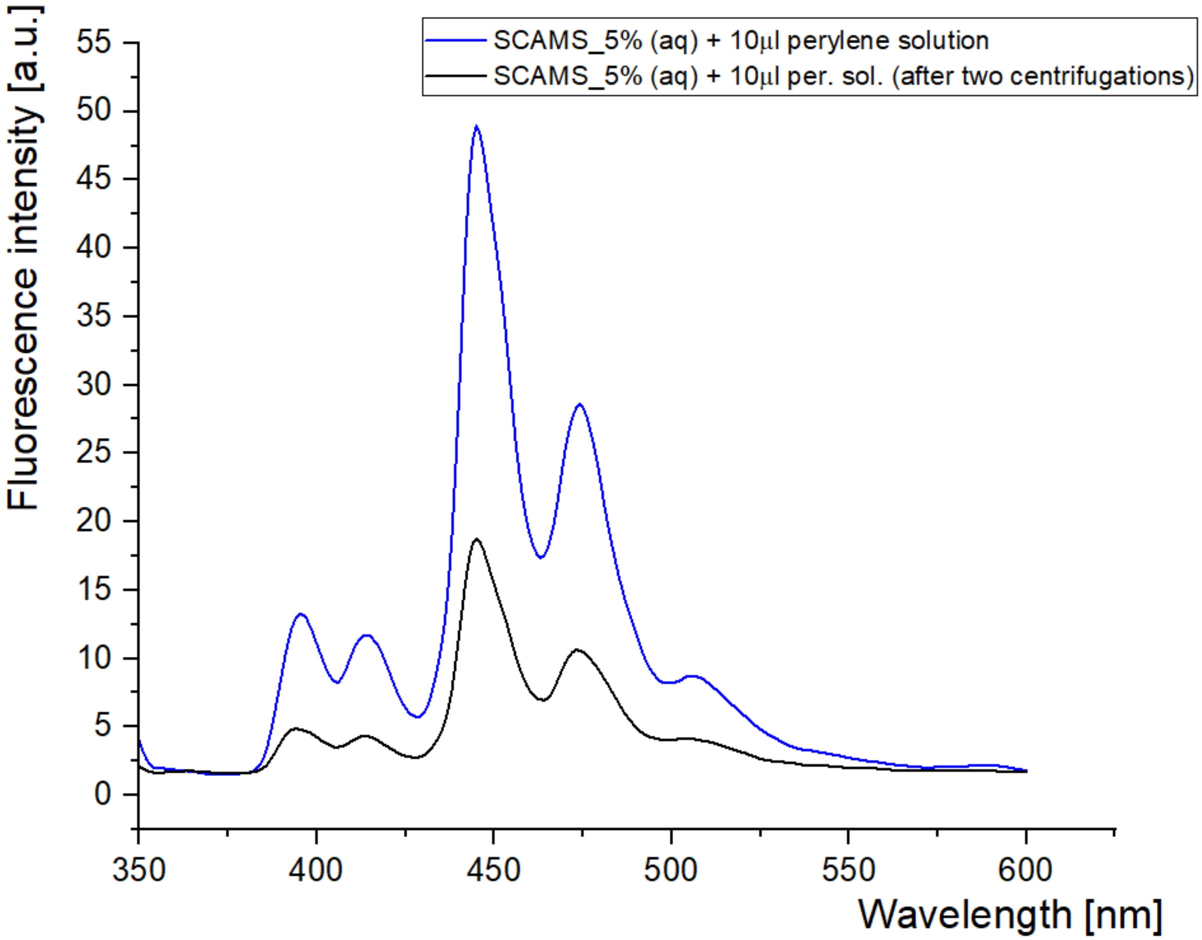

Figure S14. Comparison of fluorescence spectra of SCAMS_5% with solubilized Pe before and after isolation and purification of those particles (λ_{ex} = 330 nm).

Calculations of FRET

The quantum yields of energy transfer (Φ_{FRET}) were calculated according to the FRET theory using the following equation:[3]

$$\Phi_{trans} = 1 - \frac{I_{DA}}{I_D}$$

where I_{DA} and I_D denote fluorescence intensities at λ_{max} of An fluorescence in the presence of an energy acceptor (Pe or Fl) and in the absence of acceptor, respectively. The experimental data were collected under identical instrumental conditions for a given pair (see Fig. 4A for the fluorescence spectra of

SCAMS_5% with and without Pe, and Fig. 5A for the fluorescence spectra of SCAMS_5% with and without Fl).

The mean distances (R) between the donors and acceptors were estimated using the following relation:[3]

$$\Phi_{trans} = \frac{R_0^6}{R^6 + R_0^6}$$

where R_0 denotes the Förster critical radius of the donor-acceptor pair obtained from the PhotochemCAD database ($R_0 \approx 6$ nm for the An-Pe pair and $R_0 \approx 7$ nm for the An-Fl pair).

---

[1] Su, G., Yang, C., Zhu, J.-J. *Langmuir* **2015**, *31*, 817–823.

[2] Kaur, H., Chaudhary, S., Kaur, H., Chaudhary, M., Jena, K. C. *ACS Appl. Nano Mater.* **2022**, *5*, 411–422.

[3] J. S. Lindsey, M. Taniguchi, D. F. Bocian, D. Holten. *Chem. Phys. Rev.* **2021**, *2*, 011302.
